# Acute effects of “Munz Floor”® fascial stretching on autonomic nervous responses assessed by heart rate variability

**DOI:** 10.3389/fphys.2026.1793941

**Published:** 2026-04-07

**Authors:** Laurent Schmitt, Alexandre Munz, Catherine Schmitt, Renaud Laurent, Gregoire P. Millet

**Affiliations:** 1 ISSUL, Institute of Sport Sciences, Faculty of Biology and Medicine, University of Lausanne, Lausanne, Switzerland; 2 Independent Researcher, Marseille, France; 3 Independent Researcher, Briançon, France; 4 CREPS de Vichy, Bellerive-sur-Allier, France

**Keywords:** autonomic nervous system, fascia, fascial stretching, heart rate variability, recovery

## Abstract

**Purpose:**

To analyze if the “Munz Floor”® fascial stretching method significantly modifies the autonomic nervous responses at rest. We tested the hypothesis of a positive influence on the parasympathetic activity.

**Methods:**

Heart rate variability (HRV) was measured in thirty three (including nine females) healthy participants during a tilt test (i.e., 5-min supine followed by 5-min standing) before (pre-) and immediately after (post-) 60 min in either a control condition and 3 days later a “Munz Floor”® session. Time-domain (heart rate, HR); root mean square of the successive differences between RR intervals, (RMSSD), non-linear (standard deviations, SD1, SD2), and frequency-domain (spectral frequencies in very low, VLF; low, LF and high bands) parameters as well as the detrended fluctuation analysis (DFAα1) were measured.

**Results:**

In supine position (SU), HR_SU_ decreased to la larger extent (−12.4% ± 7.6% vs. −3.8% ± 4.0%, p < 0.001) in the “Munz Floor”® group (62.3 ± 8.7 vs. 54.4 ± 7.3 bpm, p < 0.001, Effect Size (ES) = 0.83) than in the control group (60.8 ± 8.7 vs. 58.4 ± 8.3 bpm, p < 0.01, ES = 0.27). Significant increases in RMSSD_SU_ (50.1 ± 30.1 vs. 73.3 ± 48.0 m, p < 0.001, ES = 0.59), HF_SU_ (1,212 ± 1,078 vs. 2,672 ± 2,388 m^2^, p < 0.001, ES = 0.72) were reported in the Munz Floor”® but not in the control group. In the standing position (ST): HR_ST_ decreased in both the Munz Floor”® (76.6 ± 11.0 vs. 68.8 ± 9.6 bpm, p < 0.001, ES = 0.62) and the control (77.2 ± 12.7 vs. 74.7 ± 11.9 bpm, p < 0.01, ES = 0.20) but the relative change was larger in the “Munz Floor”® (−9.6% ± 9.4% vs. −2.9% ± 6.4%, p < 0.01). Significant increases in RMSSD_ST_ (29.5 ± 23.8 vs. 39.9 ± 27.5, p < 0.01, ES = 0.38) and in (LF + HF)_ST_ (2,132 ± 2,464 vs. 3,065 ± 3,382 m^2^, p < 0.01, ES = 0.31) were observed only in the “Munz Floor”® group.

**Conclusion:**

The “Munz Floor”® fascial stretching method was effective for acutely increasing the parasympathetic activity. These results suggest “Munz Floor”® fascial stretching as a potential strategy for improving recovery and reducing the impact of stress and fatigue.

## 1 Introduction

Fascia is a connective tissue that forms a “soft skeleton” within the body. It is composed primarily of collagen fibers and plays a crucial role in support, protection, and mobility. Under conditions such as stress, fatigue, aging, poor posture, lack of movement, or injury, fascia may become rigid and adherent, thereby restricting mobility and potentially causing pain. Fascial stretching has been extensively documented in the literature (Warneke et al., 2024; Sugino et al., 2023; Ruiz et al., 2023; Siriphorn and Eksakulkla, 2020; Freitas, 2018; Flanigan et al., 2007). The primary objective is to release adhesions and rehydrate the tissues. Fascial stretching is generally gentle and dynamic; it does not involve forcing a static position, but rather employs fluid, multidirectional movements to stimulate the tissues. These movements aim to elongate long myofascial chains rather than targeting a single muscle. Several techniques may be employed, including practices inspired by yoga (Gracovetsky, 2018) or Pilates (Gouveia et al., 2022; Adiguzel et al., 2023), as well as the use of tools such as massage rollers (foam rolling) (Kerautret et al., 2021; Yoshimura et al., 2024; Konrad et al., 2021) or balls to apply targeted pressure to areas of tension. Deep breathing is often integrated, as it is essential for relaxing the nervous system and releasing deep-seated tension (Aranberri-Ruiz et al., 2022; Telles et al., 2014).

A new method named Munz Floor”® method is a floor-based exercise technique integrating isotonic, spiral movements performed at very slow velocity. Developed by dancer and choreographer Alexandre Munz, the approach aims to reduce chronic back pain and enhance postural alignment through targeted mobilization of the spinal column. The spiral sequences and alternating contraction–release cycles act as a form of deep self-myofascial release, facilitating muscular relaxation and fascia decompression. A typical session (∼60–90 min) comprises preparatory breathing and body-awareness exercises, followed by structured spiral movements emphasizing the deep paraspinal and abdominal musculature.

HRV is represented by fluctuations in the time intervals, measured in milliseconds, between successive heartbeats, also referred to as RR intervals of the electrocardiogram (ECG) complex or inter-beat intervals (IBI). HRV is considered a key indirect indicator of the autonomic nervous system (ANS) regulation, that has important effect on cardiovascular health (Bodapati et al., 2017; Arakaki et al., 2023), stress resilience (Wu et al., 2025; Tomes et al., 2025; Rudics et al., 2025; LeBlanc et al., 2025) as well as physical performance (Tekin et al., 2025; Hsu et al., 2025; Zhao et al., 2024; Freiberger et al., 2024; DeBlauw et al., 2023; Carrasco-Poyatos et al., 2022; Schmitt et al., 2006; Uusitalo et al., 2000; Schmitt et al., 2021; Schmitt et al., 2018) and the body’s adaptive capacity to external and internal changes. Its analysis provides insight into underlying physiological mechanisms and allows optimization of biological functioning (Force, 1996; Amekran et al., 2025; Sammito et al., 2024; Gronwald et al., 2024). Through HRV analysis, it is possible to investigate ANS activity and to non-invasively assess its level of activation, including parasympathetic and sympathetic energy states (Force, 1996; Amekran et al., 2025; Sammito et al., 2024; Akselrod et al., 1981; Pomeranz et al., 1985).

To date, only a limited number of studies have investigated the effects of fascial stretching on HRV and, consequently, on ANS activity. Güngör et al. (Güngör et al., 2022) reported changes in RMSSD following dynamic stretching, although these alterations were not significantly different from those observed with other recovery modalities. Beardsley CaŠ and Škarabot (2015) further indicated that self-myofascial release interventions may improve time-domain HRV parameters, suggesting a positive modulation of ANS activity. However, they emphasized considerable heterogeneity across protocols and outcomes, underscoring the need for methodological standardization. Other investigations have focused on frequency-domain indices of HRV. Borges et al. (2018) reported increased parasympathetic activity (HF) and a decrease in the LF/HF ratio after myofascial techniques, although their study primarily examined spinal manipulation rather than dynamic stretching.

The duration and persistence of dynamic stretching effects on HRV remain poorly documented. Thomas et al. (2021), in a meta-analysis, noted that cardiovascular responses to stretching may differ between acute and longitudinal interventions, though their observations largely concerned general muscular stretching rather than specific fascial techniques. Gordon et al. (2024) highlighted that distinct fascial manual therapies may elicit differential autonomic modulations, with some techniques appearing more effective in stimulating parasympathetic activity, although underlying mechanisms remain unclear. Thomas et al. (2021) also stressed that methodological variability complicates comparisons of cardiovascular effects across intervention types, a limitation particularly relevant to fascial stretching where standardized protocols are often lacking. Taken together, the available evidence indicates that fascial interventions are capable of modulating HRV, with a consistent trend toward enhanced parasympathetic modulation. Comparisons across techniques suggest variable autonomic responses depending on method and application context. Reported limitations include heterogeneity of intervention protocols, absence of controlled study designs, lack of longitudinal follow-up, and the need for methodological standardization.

We hypothesized that the Munz Floor”® fascial stretching method would significantly increase the parasympathetic energy, through enhancement of the total HRV power, HF and RMSSD, reflecting parasympathetic modulation, accompanied by a reduction in HR. These effects are expected to differ significantly from those observed during an equivalent-duration supine rest condition in the same subjects.

## 2 Methods

### 2.1 Subjects

Thirty three healthy participants were enrolled in the study with nine women (mean ± standard deviation, height 163 ± 4 cm; weight 59.1 ± 6.2 kg; age 43.2 ± 14.7 years) and 24 men (height 176.5 ± 5.8 cm; weight 74.1 ± 6.3 kg; age 37.5 ± 17.6 years).

All participants provided written informed consent to participate in the study. The study was approved by the local ethical committee (CER-VD 2024–00370) and conforms to the Declaration of Helsinki.

### 2.2 Experimental design

Participants performed two separate sessions interspersed by 72 h.

First, in late morning, all 33 subjects remained in a supine resting position without movement for 60 min. HRV was recorded immediately before and after the 60-min rest period.

During the second session, 3 days later, at the same time of day and under identical conditions, the same 33 subjects completed a Munz Floor”® fascial stretching session. This session was conducted by the voice in video-conference, without any physical contact, by the same expert (AM). HRV analysis was performed immediately before and after the Munz Floor”® session.

### 2.3 Heart rate variability

The protocol of HRV tests is presented in detail in previous articles (Schmitt et al., 2013; Schmitt et al., 2015). Briefly, the HRV test consisted of a 10-min RR interval recording at rest with 5 min supine (SU) followed by 5 min standing (ST). HRV analyses were performed on RR intervals between the 1th and 5th min supine, and between the 6th and 10th min standing. Measurement of the interval duration between 2 R waves of the cardiac electrical activity was performed with a HR monitor (Polar H10®, Kempele, Finland). Then mathematical analysis was performed with temporal (HR, RMSSD), spectral (VLF, LF, HF frequencies), non-linear (SD1, SD2) and fractal (DFAα1) analysis. The analysis and spectral power by Fast Fourier Transform (FFT), were performed with the software (Kubios®, Kuopio, Finland). The power of spectral density was measured by frequency bands in ms^2^. Hz^-1^ and the spectral power was expressed in ms^2^ (Force, 1996). The HF power band (0.15–0.40 Hz) reflects alteration of the parasympathetic influence on the heart and is related to the respiratory sinus arrhythmia (Pomeranz et al., 1985). While the LF power band (0.04–0.15 Hz) is also driven by parasympathetic tone, and presently considered responsible for carrying vagal resonances to either changes in vasomotor tone (often sympathetic) or in central modulation of sympathetic tone (Reyes del Paso GAL et al., 2013). The spectral power in the LF power band has also been shown to be related to fluctuations of arterial blood pressure (Akselrod et al., 1981; Pomeranz et al., 1985) and to baroreflex activity (Goldstein et al., 2011). Both in supine (SU) and in standing (ST) positions, LF and HF were calculated in absolute spectral power units (ms^2^) and in normalized units (nu) with LF (nu) = LF/(LF + HF) x 100 and HF(nu) = HF/(HF + LF) x 100. The total spectral power (TP) was calculated by adding LF and HF.

### 2.4 Data analysis and statistics

Data are reported as mean and standard deviation (SD). Data were tested for equality of variance (Fisher-Snedecor F-test) and for normality (Shapiro-Wilk test). When both conditions were met, a two-way repeated measures ANOVA [condition (Control, Munz) vs. measurement (pre, post)] were performed with pairwise multiple comparison procedures (*post hoc*, Tukey method). Differences in percentage changes between the conditions were tested with a Wilcoxon signed rank sum test. When either equality of variance or normality were not satisfied, variables were analysed for each condition using a Friedman test for repeated measures to determine time effects using pairwise multiple comparison procedures (Bonferroni test). In this case, differences between the Control, and Munz Floor”® condition at baseline (Pre) were tested using a Mann-Whitney rank sum test. The effect size (ES) was also calculated. Null hypotheses were rejected at P < 0.05. All analyses were completed using SigmaStat 3.5 software (Systat Software, San Jose, CA).

## 3 Results

**FIGURE 1:**
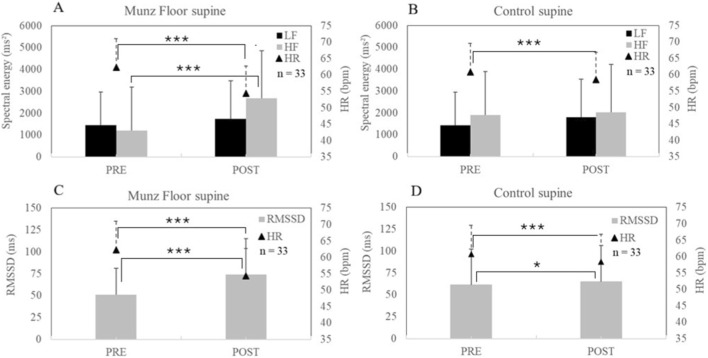
In supine position, between pre- and post-, in control (CONT) and MUNZ FLOOR® groups, mean (±SD) of heart rate (HR), heart rate variability spectral parameters [**(A,B)** with Low (LF) and High (HF) frequency bands] and root mean square of the successive differences between RR intervals [**(C,D)** with RMSSD]. * for P < 0.05, ** for P < 0.01, ***for P < 0.001 for difference between Pre- and Post-.

**FIGURE 2:**
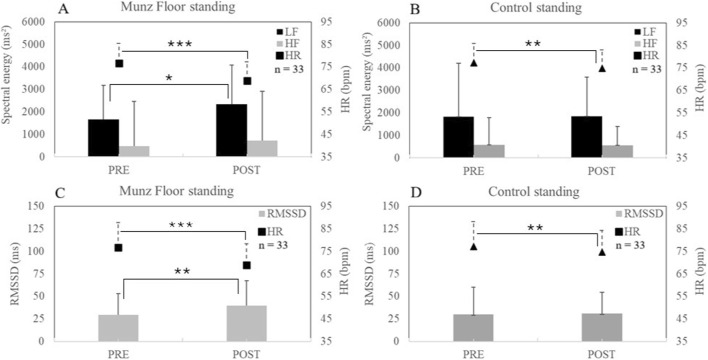
In standing position, between pre- and post-, in control (CONT) and MUNZ FLOOR® groups, mean (±SD) of heart rate (HR), heart rate variability spectral parameters [**(A,B)** with Low (LF) and High (HF) frequency bands] and root mean square of the successive differences between RR intervals [**(C,D)** with RMSSD]. * for P < 0.05, ** for P < 0.01, ***for P < 0.001 for difference between Pre- and Post-.

All the results are shown in Table 1 and Figure 1 and in Table 2 and Figure 2.

**TABLE 1:** In supine position between pre- and post-, in control (CONT) and MUNZ FLOOR® groups, heart rate (HR) and heart rate variability parameters with root mean square of the successive differences between RR intervals (RMSSD), non-linear (standard deviations, SD1, SD2), frequency-domain (very low, VLF; low, LF and high HF bands), and detrended fluctuation analysis (DFAα1).

Supine	CONT			MUNZ FLOOR®		
	Pre	Post	Δ%	Pre	Post	Δ%
HR_SU (bpm)_	60.8 ± 8.7	58.4 ± 8.3^**^	−3.8 ± 4.0^**^	62.3 ± 8.7	54.4 ± 7.3^***###^	−12.4 ± 7.6^***###^
RMSSD_SU (ms)_	61.4 ± 40.7	65.0 ± 41.5^*^	8.0 ± 13.5^**^	51.0 ± 30.1	73.3 ± 48.0^***###^	54.9 ± 37.6^***###^
VLF_SU (ms²)_	2,958 ± 3,109	3,587 ± 4,022	44.1 ± 89.9	2,332 ± 2,903	2,478 ± 2,519	50.5 ± 109.0
LF_SU (ms²)_	1,436 ± 1,516	1798 ± 1743	58.5 ± 117.2	1,448 ± 1,463	1729 ± 1,665	70.6 ± 158.6
HF_SU (ms²)_	1903 ± 1985	2024 ± 2,191	6.5 ± 30.9	1,212 ± 1,078	2,672 ± 2388^***###^	173 ± 164^***###^
LF/HF_SU_	1.7 ± 2.4	2.0 ± 3.0	55.8 ± 96.1	2.0 ± 2.3	0.9 ± 0.7^**###^	−24.9 ± 71.7^**###^
(LF + HF)_SU (ms²)_	3,339 ± 2,762	3,822 ± 3,380	17.9 ± 37.0	2,660 ± 2060	4,401 ± 3412^***##^	96.8 ± 127.9^***##^
SD1_SU_	43.8 ± 28.6	46.7 ± 29.5	8.7 ± 16.8	36.1 ± 20.9	52.4 ± 28.6^***###^	54.3 ± 37.6^***###^
SD2_SU_	94.2 ± 43.2	104.1 ± 48.9	12.0 ± 19.5^*^	88.7 ± 40.5	102.0 ± 42.3^*^	22.1 ± 34.9^*^
SD2/SD1_SU_	2.5 ± 0.9	2.6 ± 0.9	3.5 ± 13.3	2.9 ± 1.2	2.2 ± 0.8^***###^	−18.7 21.9^***###^
LF_SU (nu)_	47.1 ± 23.8	52.5 ± 21.0^*^	28.4 ± 54.4	54.7 ± 22.1	41.1 ± 19.4^***###^	−16.3 ± 48.1^***###^
HF_SU (nu)_	52.9 ± 23.8	47.5 ± 21.0^*^	−7.6 ± 20.7	45.3 ± 22.1	58.9 ± 19.4^***###^	58.1 ± 92.3^***###^
DFAα1_SU_	1.1 ± 0.2	1.1 ± 0.3	−2.8 ± 9.8	1.1 ± 0.2	1.0 ± 0.3^**^	−12.3 ± 21.3*^#^

*P < 0.05, **P < 0.01, ***P < 0.001 between Pre- and Post-, and #P < 0.05, ##P < 0.01, ###P < 0.001 in relative difference (%) between CONT and MUNZ FLOOR® change.

**TABLE 2:** In standing position between pre- and post-, in control (CONT) and MUNZ FLOOR® groups, heart rate (HR) and heart rate variability parameters with root mean square of the successive differences between RR intervals (RMSSD), non-linear (standard deviations, SD1, SD2), frequency-domain (very low, VLF; low, LF and high HF bands), and detrended fluctuation analysis (DFAα1).

Standing	CONT			MUNZ FLOOR®		
	Pre	Post	Δ%	Pre	Post	Δ%
HR_ST (bpm)_	77.2 ± 12.7	74.7 ± 11.9^**^	−2.9 ± 6.4^**^	76.6 ± 11.0	68.8 ± 9.6^***##^	−9.6 ± 9.4^***##^
RMSSD_ST (ms)_	30.1 ± 30.3	31.1 ± 23.5	17.6 ± 37.8	29.5 ± 23.8	39.9 ± 27.5^**##^	45.8 ± 49.4^**##^
VLF_ST (ms²)_	1808 ± 2,364	1,655 ± 1553^*^	41.1 ± 86.4^*^	1,491 ± 1,590	3,152 ± 4535^*#^	143.3 ± 228.2^*#^
LF_ST (ms²)_	1818 ± 2,378	1950 ± 1740	40.5 ± 94.0	1,654 ± 1842	2,344 ± 2990^*^	54.1 ± 103.6
HF_ST (ms²)_	584 ± 1,208	558 ± 828	62.0 ± 165.9	478 ± 822	721 ± 922	115.9 ± 162.4
LF/HF_ST_	10.3 ± 11.5	9.4 ± 10.8	20.8 ± 114.4	10.0 ± 13.1	8.5 ± 11.4	29.4 ± 187.9
(LF + HF)_ST (ms²)_	2,402 ± 3,505	2,408 ± 2,446	37.4 ± 77.1	2,132 ± 2,464	3,065 ± 3382^**^	62.2 ± 90.2^**^
SD1_ST_	24.3 ± 21.9	25.8 ± 17.4	20.2 ± 37.9	20.9 ± 16.8	28.3 ± 19.5^**^	45.7 ± 48.7^**#^
SD2_ST_	83.4 ± 43.7	90.0 ± 37.7^*^	15.0 ± 25.4^*^	77.2 ± 38.7	102.2 ± 46.9^***^	39.4 ± 41.7^***##^
SD2/SD1_ST_	4.4 ± 1.6	4.2 ± 1.4	−1.2 ± 17.9	4.5 ± 1.4	4.5 ± 2.0	5.1 ± 60.8
LF_ST (nu)_	81.1 ± 13.7	81.0 ± 13.7	1.0 ± 15.4	79.4 ± 14.4	75.6 ± 18.7	−4.7 ± 20.6
HF_ST (nu)_	18.9 ± 13.7	19.0 ± 13.7	19.5 ± 76.9	20.6 ± 14.4	24.7 ± 18.7	45.6 ± 96.6
DFAα1_ST_	1.5 ± 0.3	1.5 ± 0.3	−0.2 ± 12.3	1.5 ± 0.3	1.4 ± 0.3	−5.5 ± 15.4

*P < 0.05, **P < 0.01, ***P < 0.001 between Pre- and Post-, and #P < 0.05, ##P < 0.01 in relative difference (%) between CONT and MUNZ FLOOR® change.

In supine position (SU), HR_SU_ decreased to la larger extent (−12.4% ± 7.6% vs. −3.8% ± 4.0%, p < 0.001) in the Munz Floor”® group (62.3 ± 8.7 vs. 54.4 ± 7.3 bpm, p < 0.001, Effect Size (ES) = 0.83) than in the control group (60.8 ± 8.7 vs. 58.4 ± 8.3 bpm, p < 0.01, ES = 0.27). Significant increases in RMSSD_SU_ (50.1 ± 30.1 vs. 73.3 ± 48.0 m, p < 0.001, ES = 0.59), HF_SU_ (1,212 ± 1,078 vs. 2,672 ± 2,388 m^2^, p < 0.001, ES = 0.72) were reported in the Munz Floor”® but not in the control group.

Similar results were also noted in the standing position (ST): HR_ST_ decreased in both the Munz Floor”® (76.6 ± 11.0 vs. 68.8 ± 9.6 bpm, p < 0.001, ES = 0.62) and the control (77.2 ± 12.7 vs. 74.7 ± 11.9 bpm, p < 0.01, ES = 0.20) but the relative change was larger in the Munz Floor”® (−9.6% ± 9.4% vs. −2.9% ± 6.4%, p < 0.01). Significant increases in RMSSD_ST_ (29.5 ± 23.8 vs. 39.9 ± 27.5, p < 0.01, ES = 0.38) and in (LF + HF)_ST_ (2,132 ± 2,464 vs. 3,065 ± 3,382 m^2^, p < 0.01, ES = 0.31) were observed only in the Munz Floor”® group.

## 4 Discussion

The main results of the present study are:

1. The Munz Floor”® method induced a large increase in parasympathetic activity in the supine position, characterized by a decreased heart rate (HR), and increases in RMSSD and HF power (Figure 1). These effects were statistically different from those observed during passive supine rest of equal duration (Table 1).
2. A significant effect of the Munz Floor”® method was also observed in the standing position (Figure 2), with a marked decrease in HR and simultaneous increases in RMSSD, LF band power, and total LF + HF power (Table 2). In the present study, LF increased in the standing position but not in the supine position. However, in standing, the LF/HF ratio did not increase, while RMSSD increased and HR decreased. Although these effects are less pronounced than in the supine position, the significant parameters observed in standing still indicate an increase in parasympathetic modulation rather than sympathetic activation.

It is commonly admitted that HR and HRV indices cannot be interpreted as interchangeable markers of parasympathetic activity, and that their concurrent evolution requires clarification. A reduction in HR does not directly quantify parasympathetic activity, as HR reflects the net effect of both sympathetic and parasympathetic influences as well as intrinsic sinoatrial node properties. However, when HR decreases simultaneously with increases in vagally mediated HRV indices such as RMSSD and HF power, the combination is generally interpreted as reflecting a shift toward greater parasympathetic dominance. This interpretation is supported by the fact that RMSSD and HF specifically index high-frequency beat-to-beat variability generated by respiratory sinus arrhythmia, which is tightly linked to cardiac vagal modulation.

HR and HRV parameters provide complementary and no redundant information. HR captures the tonic output of autonomic influences on the sinoatrial node, whereas RMSSD and HF reflect phasic vagal modulation. Therefore, the concurrent decrease in HR and increase in vagally mediated HRV indices in our study suggest both a reduction in overall cardiac chronotropic drive and an enhancement of beat-to-beat vagal modulation (Force, 1996; Sammito et al., 2024; Quigley et al., 2024).

The difference in outcomes between the control group (supine rest) and the experimental group (Munz Floor”®) arises from the additional mobilization performed during the intervention. This superior effect on parasympathetic energy results from the combination of three important components of the Munz Floor”® session (i.e., voice; slow spiral-like movement; pain modulation) that are detailed below:

The session is guided by the voice: the tone and slow pace of speech, interspersed with pauses, create a condition resembling meditation. This reduces tension and induces deep relaxation. Meditation and mindfulness are well-documented to enhance parasympathetic activity (Pascual et al., 2023). The slowness of both voice and movement reduces respiratory frequency (RF), which contributes to increased parasympathetic activity. de Souza Neto EPN and Lehot (2003) and Laborde et al. (2022) demonstrated that lowering RF promotes parasympathetic activation.

Participants lie on the floor and perform extremely slow, spiral-like movements aimed at stretching the fascia. Several studies have shown that fascial interventions can significantly modulate HRV, with a tendency toward improved parasympathetic activity. de Sousa et al. (2020) reported that manual fascial therapies can induce changes in physiological markers of autonomic modulation, suggesting possible involvement of anti-inflammatory and vagal pathways. These findings are supported by Ruffini et al. (2015), who documented significant parasympathetic activation following fascial interventions. Güngör et al. (2022) observed changes in RMSSD after dynamic stretching, although these modifications were not significantly different from those induced by other recovery methods. Beardsley CaŠ and Škarabot (2015), in their meta-analysis, reported that self-myofascial release interventions can improve time-domain HRV parameters, suggesting positive modulation of ANS activity. Borges et al. (2018) demonstrated increased parasympathetic activity (HF) and decreased LF/HF ratio after myofascial techniques, although their study focused on spinal manipulation rather than dynamic stretching specifically. These observations were confirmed in a meta-analysis (de Sousa et al., 2020), showing that manual fascial therapies can induce immediate increases in parasympathetic activity, reflected in changes in HRV frequency components. Nevertheless, they also emphasized the scarcity and heterogeneity of studies specifically addressing stretching. Comparisons across techniques suggest variable autonomic responses depending on the method and application context, with few direct comparative studies available.

The present results contribute to filling this gap by demonstrating the effects of spiral fascial stretches performed slowly on the floor, guided by voice, on parasympathetic activity of the ANS. This effect was observed not only in the supine resting position but, more surprisingly, also in the standing position. While in the supine position homeostatic regulation predominates, with expected parasympathetic dominance, in the standing position baroreceptors are engaged to regulate peripheral pressures and facilitate venous return to the heart and brain, thereby increasing sympathetic activity and reducing parasympathetic influence. It is therefore noteworthy that in this standing position, the Munz Floor”® method activated the parasympathetic activity but to a less extend than in supine position (Figure 2).

Several physiological mechanisms underlying the effects of myofascial techniques have been proposed: they involve complex interactions between neuromuscular, proprioceptive, and autonomic systems, with an important role for mechano-transduction and viscoelastic modulation of fascial tissues. Beardsley CaŠ and Škarabot (2015) emphasized that changes in fascial mechanical properties can influence force transmission and mechano-signaling, potentially affecting cardiac autonomic regulation. Thomas et al. (2021) also noted that peripheral hyperaemic responses and the release of vasoactive substances following stretching may contribute to cardiovascular adaptations, although these mechanisms have mainly been studied in the context of general muscle stretching. Borges et al. (2018) highlighted the importance of cervical afferents and vestibular inputs in autonomic modulation following fascial interventions. These neurophysiological pathways involve complex interactions between fascial mechanoreceptors and the ANS. The authors suggest that mechanical stimulation of fascial tissues may activate neurovegetative reflexes via these afferent pathways. Beardsley CaŠ and Škarabot (2015) further expanded this understanding by describing the role of mechano-transduction on fibroblasts and the neuromodulator inhibition of pain through mechanoreceptor activation. These mechanisms may directly influence autonomic regulation and, consequently, HRV.

The relationship between HRV and pain is embedded within integrative models of central regulation, notably the Neurovisceral Integration Model (NVIM). This model posits that a central autonomic network (CAN), comprising the insula, anterior cingulate cortex, amygdala, prefrontal cortex, and brainstem, orchestrates both autonomic flexibility (HRV) and pain regulation. Cocoli et al. (2025) demonstrated that vagus nerve stimulation activates afferent and efferent fibers, the nucleus tractus solitarius (NTS), the locus coeruleus, the amygdala, and the prefrontal cortex. Anti-inflammatory effects are mediated through suppression of pro-inflammatory cytokines (TNF-α, IL-1β, IL-6) and activation of α7nAChR receptors on macrophages. Vagus nerve stimulation reduces pain via descending serotonergic, noradrenergic, and cholinergic pathways, as well as through attenuation of central and peripheral inflammation.

Elevated parasympathetic activity is so correlated with enhanced pain inhibition and reduced nociceptive perception, regardless of the mode of pain induction. Forte et al. (2022), in a meta-analysis of 71 studies (n = 6,364), reported the relationship between parasympathetic activity and pain perception during experimental stimulations (cold, heat, pressure, electrical, visceral). Results showed that increased parasympathetic activity (HF, RMSSD) was associated with greater pain inhibition capacity and reduced perceived intensity. Pellow et al. (2025) compared non-invasive vagus nerve stimulation (nVNS) and HRV biofeedback (HRVB) in chronic pain. In a sample of 813 subjects, nVNS significantly reduced the frequency and intensity of headaches/migraines, decreased medication use, and improved quality of life. HRVB reduced pain and depression while increasing parasympathetic energy. Daibes et al. (2025), in a meta-analysis of 23 studies (n = 1,262), indicated that pain-relief interventions (including VNS, HRVB, TENS, acupuncture) significantly increased parasympathetic indices (RMSSD, HF), with a reduction in the LF/HF ratio, reflecting a shift toward parasympathetic dominance. Interventions that activate the parasympathetic system reduce pain, confirming the central role of parasympathetic activity in nociceptive modulation.

Thus, the Munz Floor”® method which show a significant increase in parasympathetic activity could be effective to reduce pain and inflammations.

## 5 Limitations

Assessing blood pressure and respiratory rate would have provided a more comprehensive analysis. We chose to rely solely on HRV measurements in order to maintain a more flexible organizational and technical setup, given the large number of participants and the limited time available for conducting the study within a clinical setup. We aimed also to run as study that may have practical application. Unlike the continuous monitoring of blood pressure or ventilatory patterns, HRV can be readily measured, even in clinical settings.

Although pain attenuation has already been reported with the “Munz Floor”® method, a direct assessment of pain modulation using validated scales or questionnaires would have strengthened the practical relevance of the findings by clarifying whether the reported increase in parasympathetic activity was associated with pain reduction.

## 6 Conclusion

The Munz Floor”® fascia stretching method was effective for increasing the parasympathetic activation. Prior interventional evidence, the present results suggest that this method may be recommended to enhance recovery in athletic populations and to mitigate the effects of stress and fatigue in the general population. The intervention can be applied repeatedly without inducing adverse physical effects and may therefore be considered a safe strategy for promoting overall health.

## Data Availability

The raw data supporting the conclusions of this article will be made available by the authors, without undue reservation.
